# Advances towards a Marker-Assisted Selection Breeding Program in Prairie Cordgrass, a Biomass Crop

**DOI:** 10.1155/2012/313545

**Published:** 2012-11-26

**Authors:** K. R. Gedye, J. L. Gonzalez-Hernandez, V. Owens, A. Boe

**Affiliations:** Department of Plant Sciences, South Dakota State University, Brookings, SD 57007, USA

## Abstract

Prairie cordgrass (*Spartina pectinata* Bosc ex Link) is an indigenous, perennial grass of North America that is being developed into a cellulosic biomass crop suitable for biofuel production. Limited research has been performed into the breeding of prairie cordgrass; this research details an initial investigation into the development of a breeding program for this species. Genomic libraries enriched for four simple sequence repeat (SSR) motifs were developed, 25 clones from each library were sequenced, identifying 70 SSR regions, and primers were developed for these regions, 35 of which were amplified under standard PCR conditions. These SSR markers were used to validate the crossing methodology of prairie cordgrass and it was found that crosses between two plants occurred without the need for emasculation. The successful cross between two clones of prairie cordgrass indicates that this species is not self-incompatible. The results from this research will be used to instigate the production of a molecular map of prairie cordgrass which can be used to incorporate marker-assisted selection (MAS) protocols into a breeding program to improve this species for cellulosic biomass production.

## 1. Introduction

Recent world issues associated with fuel consumption and supply have turned attention towards biofuel production, especially cellulosic biofuel. Perennial grasses provide an optimal source of cellulosic biomass due to their high yield potential. Prairie cordgrass (*Spartina pectinata* Bosc ex Link) is a perennial indigenous grass of North America and can be found as a native from Texas to near the Arctic Circle [1]. Ongoing studies on prairie cordgrass in comparison with switchgrass (*Panicum virgatum* L.) indicate that prairie cordgrass could produce more biomass than switchgrass [2]. Furthermore, results from the comparison of prairie cordgrass and switchgrass performed by Boe and Lee in 2007 [2] indicated that prairie cordgrass has a wider environmental amplitude and is adapted to poorly drained wet areas which can have high salinity and be poorly aerated, regions not suitable for the production of conventional crops such as maize (*Zea mays*) [2, 3]. These results are indicative of the potential of prairie cordgrass as a source of biomass for cellulosic biofuel production.

A research program at South Dakota State University (SDSU) is underway to develop native prairie cordgrass into a viable cellulosic biomass crop. The development of a new crop species requires a multidisciplinary approach; examining and validating each step before commercialization can occur. These steps include, but are not limited to, the assembling of a germplasm collection, the development of an accelerated breeding program, and the optimization of cultivation practices. A fundamental step to accelerate the breeding of prairie cordgrass is to determine and optimize a crossing protocol. prairie cordgrass has been intrinsically believed to be a protogynous outcrossing species, based on the mode of reproduction of its maritime relative smooth cordgrass (*Spartina alterniflora*) [4, 5]. From work performed upon other members of the *Spartina* genus, it has been conjectured that prairie cordgrass will have a similar method of reproduction. In the majority of other Graminaceae species, breeding is performed via the initial emasculation of the floret to ensure that only cross pollination can occur. Prairie cordgrass has an inflorescence composed of between 0 and 31 short paracladia and 11–13 long paracladia [6], bearing a total of 10–80 fertile spikelets, of single florets [7]. Physical emasculation of prairie cordgrass is essentially impractical, but this technique may not be necessary as with protogyny and ascertaining the appropriate timing, directed cross pollination is feasible. Self pollination has not been identified previously in prairie cordgrass and assumptions have been made that prairie cordgrass may be self incompatible, if this is the case then the classic mapping technique of producing recombinant inbred lines will be fundamentally impossible and alternate strategies will need to be utilized.

An initial stage of the prairie cordgrass project is to develop a molecular map of the species. Only limited molecular analyses have been performed upon prairie cordgrass, notably in contrast to its maritime relative *S*. *alterniflora*. In the National Centre for Biotechnology Information (NCBI), only 57 prairie cordgrass sequences have been deposited (predominantly regions of the nuclear and organelle genomes utilized for diversity analysis, that is, *Waxy* (AY508655 and AF372461), ITS (AF019843, AJ489796, and EF153082) and the chloroplast *trnL-trnF* intergenic spacer region (EF137568, EU056305, and AF372625)). A recent publication on the analysis of the transcriptome of prairie cordgrass has increased the available knowledge on the genome of the species [8]. Constructed from the analysis of the expression sequence tags (ESTs) and identified from the transcriptome of prairie cordgrass, a total of 26,302 contigs and 71,103 singletons were assembled with all sequence information available as supplemental data [8]. Additional molecular analysis of genetic diversity of prairie cordgrass in natural populations in Minnesota with amplified fragment length polymorphism (AFLP) has been performed [9].

An optimal marker system for the development of a molecular map of prairie cordgrass are SSRs or microsatellites. Microsatellites are highly utilized molecular markers and have been developed for the majority of agronomically and economically important crop species [10]; their efficacy arises predominantly due to their reproducibility, codominant inheritance, and abundance [11]. Modern techniques allow the rapid identification of microsatellite regions in species which have limited sequence information, and by using proprietary genomic library screening techniques, microsatellites have been developed for numerous non-model organisms, encompassing insects [12], birds [13], fish [14], mammals [15], and plants, including other species in the *Spartina* genera [16]. Previous studies on other members of the *Spartina* genera have developed 35 markers for microsatellite loci in *S*. *alterniflora* [16, 17]. Of these *S*. *alterniflora* markers, three have been found to amplify in prairie cordgrass [16]. SSRs have been used extensively in numerous crop species, in wheat (*Triticum aestivum* L.), and another Poaceae species; SSRs have been used to enhance molecular maps begun with restriction fragment length polymorphism (RFLP) and to identify genes of interest [18]. Furthermore, Wilde et al. in 2007 [19] found that the incorporation of MAS with SSR markers into a traditional breeding program can result in a substantial increase in the incorporation of *Fusarium* head blight resistance, a quantitative trait, in wheat with the shortest possible time. The results of Wilde et al. [19] are indicative of the potential of MAS for the breeding of prairie cordgrass.

This paper details the first investigation into initial breeding and crossing of prairie cordgrass, specifically the production of F_1_ individuals and the validation of a crossing protocol. Furthermore, the characterization of 35 microsatellite loci in prairie cordgrass from genomic DNA is discussed. The validation of the microsatellite loci occurred in a prairie cordgrass germplasm collection and a reciprocal cross as an initial step in the development of a molecular map, which can be further utilized during marker-assisted selection of lines with traits desirable for the improvement of prairie cordgrass as a biofuel crop.

## 2. Materials and Methods

### 2.1. Genomic Library Construction

Genomic DNA was extracted from four lines of prairie cordgrass using the method as described by Karakousis and Langridge in 2003 [20], with minor modifications. A mixture of the four random prairie cordgrass clones DNA, totaling > 100 *μ*g, was sent to Genetic Identification Services (GIS) (http://www.genetic-id-services.com/) for the development of genomic libraries. The genomic libraries were enriched for the four simple sequence repeat (SSR) motifs (CA)_*n*_, (GA)_*n*_, (AAG)_*n*_, and (CAG)_*n*_. Subsamples of 100 clones were sequenced and primers were designed to flank the SSR regions using DesignerPCR, version 1.03 (Research Genetics, Inc.) and PRIMER 3 [21]. The SSR regions were classified according to Jones et al. [22] as being pure repeats (i.e., [N_1_N_2_]_*X*_), compound repeats (i.e., [N_1_N_2_]_*X*_[N_3_N_4_]_*Y*_), and interrupted repeats (i.e., [N_1_N_2_]_*X*_N_3_[N_4_N_5_]_*Y*_).

### 2.2. Plant Material

Wild germplasm has been collected from throughout the mid-western states of the United States, creating a core germplasm collection to be utilized in prairie cordgrass breeding (unpublished data). A geographically diverse sample of sixteen plants, collected from South Dakota, North Dakota, Minnesota, and Iowa, was characterized using the identified SSR loci. A sample from the closely related species *S*. *spartinae* was also included to examine cross species amplification. The germplasm collection was grown in standard greenhouse conditions.

**Figure 1:**
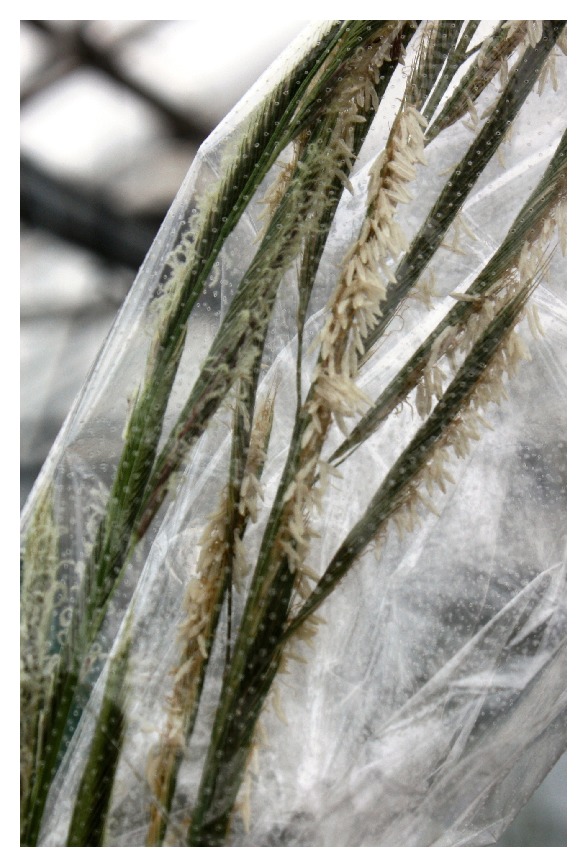
Two prairie cordgrass heads undergoing crossing, contained within a single bag.

Crosses between and amongst these genotypes were performed. Crossing between two plants of prairie cordgrass was performed in the following manner; two inflorescence at the appropriate stage of development were placed inside a crossing bag for seven days, producing F_1_ plants (Figure 1). A reciprocal cross between two genotypes (designated RR2 and RR21) from the Red River morphotype was produced. The reciprocal cross produced 45 F_1_ plants from the RR21 × RR2 directed cross and 49 F_1_ plants from the RR2 × RR21 directed cross, a total of 94 plants. Two genotypes identified as clones (designated SP1.1B and SP1.2A) from the SSR evaluation were crossed via the same technique, producing a total of 46 F_1_ individuals; a total of 20 F_1_ plants were produced when SP1.1B was used as the maternal parent and 26 F_1_ plants when SP1.2A was used as the maternal parent. All F_1_ plants and their respective parents were grown in standard greenhouse conditions.

### 2.3. DNA Extraction and Evaluation of SSR Primers

DNA was extracted from all examined plants using the method as described by Karakousis and Langridge in 2003 [20], with minor modifications. Evaluation of the primers was performed in a PCR reaction consisting of 2 U *Taq* DNA polymerase (Promega GoTaq), 1 × PCR Buffer (Promega GoTaq), 2 mM MgCl_2_, 0.6 mM dNTPs, and 0.6 mM of each primer, with a final volume of 20 *μ*L [23]. PCR reactions were performed in a BioRad MyCycler (BioRad, Hercules, CA). Initially a gradient of annealing temperatures was used to determine optimal *T*_*a*_, as these primers were designed to be utilized in a prairie cordgrass breeding program, a robust and high-throughput protocol was developed. Specific thermocycling conditions for the primers used in this study were as follows: an initial denaturation at 94°C for 5 minutes, followed by 35 cycles of 94°C for 1 minute, 53 or 55°C for 1 minute, 72°C for 1 minute, followed by an extension step of 72°C for 10 minutes, and a 10°C hold. PCR product was visualized on 8% nondenaturing PAGE; bands were scored and sized by comparison to a 100 bp ladder using AlphaEaseFC Software (Alpha Innotech, San Leandro, CA).

### 2.4. SSR Markers Analysis

Polymorphic bands in the two examined populations described above were scored on a presence (1) or absence (0) basis. An estimation of genetic distance was calculated with Nei and Li's algorithm [24] and the resulting matrix was clustered with unweighted pair group method with arithmetic mean (UPGMA) [25]. Analysis was performed using multivariate statistics package (MVSP) [26].

## 3. Results

### 3.1. Germplasm and Crossing

**Figure 2:**
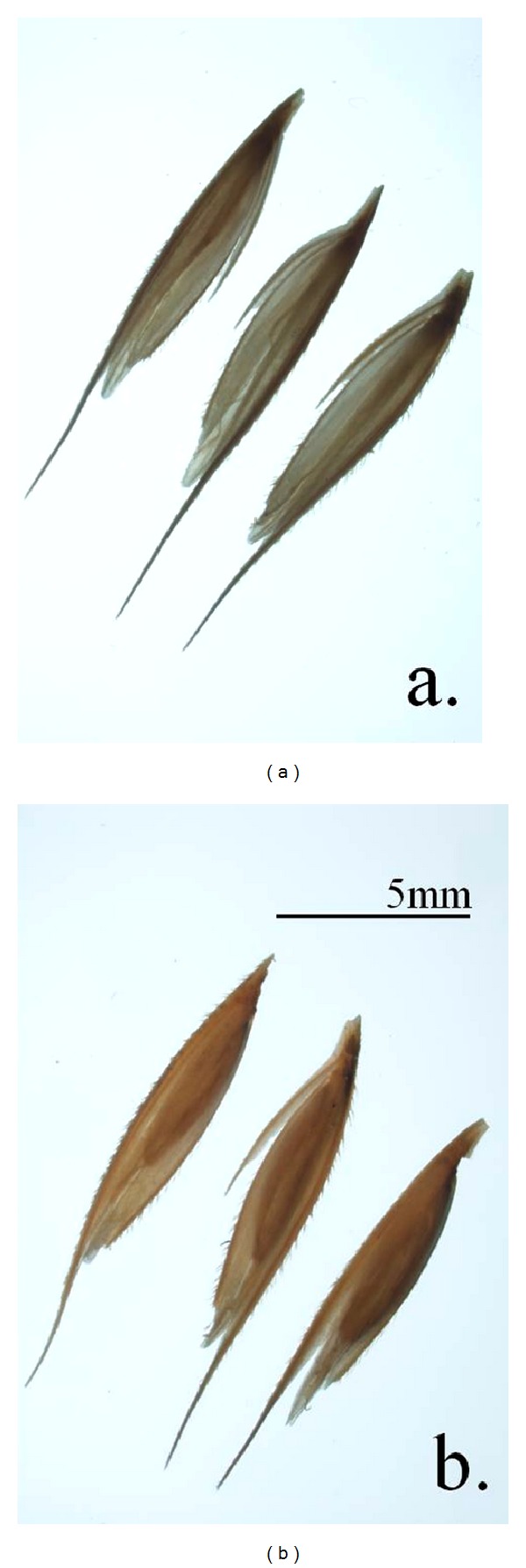
Prairie cordgrass seeds photographs taken over a light box. Illuminating prairie cordgrass with light box is used to distinguish the presence of a developed embryo in the seeds. (a) Non-viable seeds. (b) Viable seeds.

**Figure 3:**
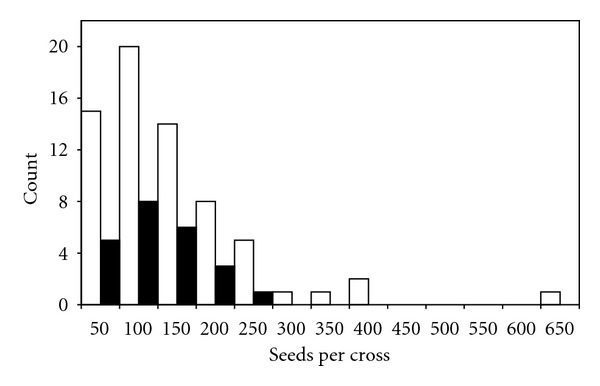
Summary graph of results of crosses between members of the core germplasm collection of prairie cordgrass. White bars indicate self crosses and black bars indicate out crosses.

The crossing of the germplasm collection was successful with the production of a total of 14,813 putatively viable seeds from 110 crosses, performed in lines and collected from 48 distinct collection locations (Table 1). Numerous nonviable seeds were produced from each cross. A seed was determined to be viable if the actual seed (endosperm and embryo) was visible within the glumes (Figure 2). The number of viable seeds produced per head varied from 6 to 642, with an average of 134 seeds per cross. The crosses can be grouped into two, dependent upon the parents, those that were outcrossed and those that were selfed. Crosses that were designated “out” were between genotypes that were geographically diverse, while crosses designated “self” were produced from members of the population collected at the same location. The average number of seeds produced from outcrossed individuals was 143, with a range of 12 to 250, while selfed individuals produced an average of 112 seeds per cross, with a range of 6 to 642 (Figure 3).

### 3.2. Microsatellite Characterization

Of the total 100 genomic clones sequenced, 70 contained microsatellite motifs, 26 from the (CA)_n_ enriched library, 25 from the (GA)_n_ enriched library, 3 from the (AAG)_n_ enriched library, and 16 from the (CAG)_n_ enriched library. All 70 loci were examined and 35 were amplified with the standardized conditions (Table 2). The repeat SSR structure that occurred most frequently was the pure repeat (27), followed by the compound repeat (7) and only one interrupted repeat sequence was found (Table 2).

Only two primers produced monomorphic profiles from the analysis of the sixteen lines, SPSD004 from the (GA)_n_ library and SPSD048 from the (AAG)_n_ library. The remaining primers produced between 1 and 12 scorable bands. Of the examined primers, 11 were amplified between 1 and 7 bands in *S*. *spartinae* (Table 2).

All sequences were examined using BLASTn [27] on the National Center for Biotechnology Information (NCBI) server for similarities to other recorded sequences. Only two of the prairie cordgrass sequences displayed homology (*e*≤*e* ^−20^) to recorded sequences, SPSD003 to six sequences from rice, wheat, and field mustard (*Brassica rapa*) (accession numbers: AP008214.1, AP005495.3, NM_001067901.1, AK111788.1, EU660901.1, and AC189183.2) and SPSD050 to five sequences all from rice (accession numbers: CR855236.1, AP008210.1, AL606607.3, NM_001059819.1, and AK108706.1) [25].

### 3.3. Crossing Population

**Figure 4:**
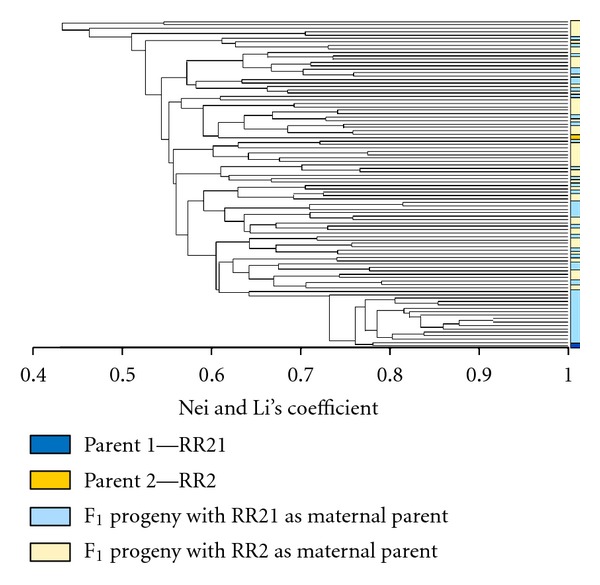
Unrooted dendogram clustered with UPGMA of the genetic association of the F_1_ progeny from a cross between prairie cordgrass lines RR21 and RR2, from the analysis of SSR regions with Nei and Li's Coefficient of genetic similarity.

A total of 94 individuals from the RR2 × RR21 and the RR21 × RR2 crosses were examined with 35 SSR primers. The amplified bands were scored based on presence or absence, the resulting data was analyzed with Nei and Li's coefficient producing a similarity matrix (data not shown), and the data was clustered with UPGMA producing a dendogram (Figure 4). The results of the dendogram indicate that each individual was a cross between the two parents. When the population derived from the cross between the two plants (SP1.1B and SP1.2A) and determined to be clones was examined, bands present in both parents were found to segregate in the progeny (data not shown), indicative of sexual recombination; this result provides validation that successful crossing occurred.

## 4. Discussion

The primary requirement of any breeding program is to ensure that accurate crosses are made; in many other members of the Poaceae this is achieved by physical or chemical emasculation. The prolific numbers of flowers per head in addition to the small size of the flowers make physical emasculation unfeasible. Furthermore, due to limited knowledge about the nature of the fertility of this species, chemical emasculation has not been developed for prairie cordgrass. The results from the SSR analysis indicate that utilizing the inherent protogyny of prairie cordgrass allows successful crossing between two individuals without the need for emasculation, confirming the validity of the breeding methodology used. The presence of individuals in the F_1_ mapping populations which show limited genetic dissimilarity from the parents could be evidence of selfing; further investigations are required. The presence of these potential selfed individuals indicates that future breeding and/or mapping populations should be examined with the molecular markers devised in this research to remove suspect individuals. Subsequently, the successful crossing between two clones is indicative that prairie cordgrass may not be self-incompatible and that it may be possible to develop in this species conventional mapping populations, such as recombinant inbred lines. Further studies into self-compatibility with investigations into potential apomictic prairie cordgrass plants are underway. The number of seeds observed in this research appears to be larger than what was previously described by Clayton et al. [7]. The variation between the two studies can be attributed to both environmental and genetic variations. Genetic variation in seed set in prairie cordgrass, although at this stage not quantified, is demonstrated by the range in viable seed set observed in this research.

The amplification of *S*. *spartinae* with the SSR primers developed in this research are indicative of the potential colinearity amongst the genomes of *Spartina* spp. and other grass species; this colinearity will allow easier identification and characterization of genes. The colinearity between prairie cordgrass and other Poaceae is currently utilized to examine genes identified in related species in prairie cordgrass, specifically genes utilized to examine phylogeny (i.e., the *waxy* gene for granule bound starch synthase).

The prevalence and distribution of SSR regions across plant genomes are extremely variable. Variation in SSRs is not limited to their location, but also their motif, putative function, abundance, and repeat number [28]. The results of this analysis indicate that the two dinucleotide repeats (CA)_n_ and (GA)_n_ are more prevalent in prairie cordgrass (31% and 46%, resp.), than the two trinucleotide repeats (AAG)_n_ and (CAG)_n_ (6% and 17%, resp.). The prevalence of the dinucleotide motif in prairie cordgrass is similar to what was observed in the characterization of SSRs in other *Spartina* sp., where Blum et al. [16] found 82% of isolated SSRs contained dinucleotide repeats and Sloop et al. [17] found 71% containing similar motifs. In all three studies the di- and trinucleotide repeats occurred as perfect, compound, and interrupted motifs. Based on the results found in this research, the genomic libraries enriched with the dinucleotide repeats have been extensively sequenced; the resulting sequence information will be screened to isolate additional SSR regions, primers will be designed, and the resulting markers will be used to develop a molecular map of prairie cordgrass. The molecular map will then be used to find linkage between SSR markers and traits of interest allowing future MAS to be performed.

## Figures and Tables

**Table 1:** Summary of crosses performed amongst the core prairie cordgrass germplasm collection. The type of cross is designated “Out” for crosses between geographically diverse members and “Self” for members of the population collected at the same location.

Female parent	Male parent	Number of crosses	Type	Total no. of seeds
SP1	SP21	2	Out	126
SP1	SP71	1	Out	71
SP21	SP30	1	Out	51
SP21	SP31	6	Out	484
SP21	SP4	2	Out	248
SP21	SP42	1	Out	78
SP21	SP66	2	Out	233
SP21	SP7	1	Out	82
SP24	SP30	1	Out	180
SP29	SP22	1	Out	146
SP29	SP30	1	Out	42
SP3	SP77	1	Out	282
SP30	SP31	1	Out	164
SP30	SP52	1	Out	246
SP31	SP21	4	Out	408
SP31	SP30	1	Out	28
SP31	SP32	1	Out	38
SP31	SP46	3	Out	211
SP31	SP5	1	Out	81
SP31	SP66	1	Out	46
SP31	SP79	1	Out	198
SP32	SP21	1	Out	173
SP32	SP31	2	Out	310
SP32	SP45	1	Out	296
SP32	SP66	1	Out	240
SP32	SP67	1	Out	193
SP32	SP78	1	Out	94
SP35	SP78	1	Out	279
SP41	SP42	1	Out	6
SP41	SP45	1	Out	86
SP44	SP54	1	Out	312
SP44	SP59	1	Out	274
SP45	SP31	1	Out	282
SP45	SP49	1	Out	225
SP45	SP67	1	Out	138
SP49	SP40	1	Out	203
SP49	SP45	1	Out	176
SP49	SP66	2	Out	237
SP5	SP31	1	Out	189
SP5	SP78	1	Out	407
SP53	SP67	2	Out	43
SP54	SP44	1	Out	145
SP54	SP59	2	Out	81
SP56	SP58	1	Out	235
SP65	SP57	1	Out	70
SP66	SP31	1	Out	93
SP67	SP32	1	Out	99
SP67	SP45	2	Out	583
SP67	SP53	1	Out	185
SP7	SP46	1	Out	160
SP72	SP1	1	Out	125
SP72	SP30	2	Out	459
SP75	SP13	1	Out	59
SP77	SP72	1	Out	163
SP77	SP79	1	Out	184
SP78	SP35	1	Out	94
SP78	SP79	1	Out	113
SP82	SP14	1	Out	642
SP21	SP21	13	Self	1470
SP22	SP22	1	Self	110
SP31	SP31	13	Self	1377
SP40	SP40	1	Self	126
SP41	SP41	2	Self	393
SP53	SP53	1	Self	12
SP74	SP74	2	Self	229

**Table 2:** Summary and description of primer sequences designed for SSR regions identified in prairie cordgrass and their amplification products.

Locus	Library	Primer sequence (5′3′)	SSR motif	Expected size (bp)	Size range (bp)	*T*_*a*_ (°C)	GenBank ID
SPSD001	(CA)_*n*_	F: GCTGCTCCTCTTCTCTGTCT	(CA)_13_	180	212–386	55	GQ354531
		R: ACGGCACACTTAGTTTTCTG	Perfect				
SPSD002	(CA)_*n*_	F: GCACTGTTTGGTGATGCTC	(CA)_13_	249	100–655	55	GQ354532
		R: CTGACGCAAGGTTGATGAG	Perfect				
SPSD009	(CA)_*n*_	F: ATGGTTTCACAAGTCGGAAGT	(GT)_15_	166	215–432	55	GQ354533
		R: CAGGGCTGCCTACAAGATG	Perfect				
SPSD010	(CA)_*n*_	F: AACCAAAAGGATAGACCCTA	(CA)_28_	168	127–310	53	GQ354534
		R: ACGAAATATGTGACCGATAC	Perfect				
SPSD011	(CA)_*n*_	F: CTAACGTATGTCGTTCATGTGG	(GT)_11_	135	147–251	53	GQ354535
		R: AAGGCGATTTTAAGAGGCTAAG	Perfect				
SPSD013	(CA)_*n*_	F: GGGATGCTTTGTAGATAAGAAA	(CA)_41_	157	85–173	55	GQ354536
		R: TCTTCCTCTTTACTCTGTCACC	Perfect				
SPSD016	(CA)_*n*_	F: CCGACTACGAGCCACATT	(CA)_14_	155	156–268	53	GQ354537
		R: GTTCCACACATACGAAGGAGA	Perfect				
SPSD019	(CA)_*n*_	F: CCTGCTTACTCTTACTCCGTC	(CA)_13_(GA)_7_	134	220–365	53	GQ354538
		R: ACCCTTTTTTCTTTTGGTCTC	Compound				
SPSD020	(CA)_*n*_	F: ATGAGACGATAGCAGGATGAC	(CA)_23_	178	146–288	55	GQ354539
		R: AGCAGATTACGATTCAGATGG	Perfect				
SPSD025	(CA)_*n*_	F: CAGTCCATGCAACTCAGAAGTA	(CT)_10_(CA)_38_	269	204–748	55	GQ354540
		R: AACCTGATAGAAGTGGTCATGC	Compound				
SPSD026	(CA)_*n*_	F: GTGGAATCAACAACACCAGA	(GA)_13_(GT)_20_	209	196–539	55	GQ354541
		R: GTCGCTTTAGCCCGTAAG	Compound				
SPSD003	(GA)_*n*_	F: ATGGAAACTGTCTGGAACTGAC	(CT)_28_	294	231–263	55	GQ354542
		R: AGCAATAACCACAGAAGAGACC	Perfect				
SPSD027	(GA)_*n*_	F: TCAAACAATGGCGGAGAAG	(CT)_17_	214	188–224	55	GQ354543
		R: CTGGCTCCACCTCTTTGG	Perfect				
SPSD004	(GA)_*n*_	F: GTTGCTCGGTTCCAGTTG	(GA)_22_	178	133 & 164	55	GQ354544
		R: CGCCACACAAAAGTAGCC	Perfect				
SPSD031	(GA)_*n*_	F: TCGCACTTTTGATTCTCTTTAC	(CT)_20_	188	155–346	53	GQ354545
		R: TGGATGGATTAGGTTACTGTTG	Perfect				
SPSD032	(GA)_*n*_	F: CTCTCGCCCATTGCTACTTA	(CT)_13_	196	146–193	53	GQ354546
		R: CCATTGCTATGTTGTTTGAGC	Perfect				
SPSD034	(GA)_*n*_	F: CAGGTCTACGGAGGTCACTAC	(CT)_12_	160	144–306	53	GQ354564
		R: TCAAAAGAAGAGCACATACACA	Perfect				
SPSD036	(GA)_*n*_	F: TTCACCACACCACTTTATCC	(CT)_29_	271	222–260	53	GQ354547
		R: GGAAGCAACAAACATTGATG	Perfect				
SPSD039	(GA)_*n*_	F: CTTTCAGATAGCTCCACTGATC	(GA)_12_	206	193–448	55	GQ354548
		R: AGCAATAACTGTGCATACCTCT	Perfect				
SPSD040	(GA)_*n*_	F: AATCGAAGTAGCAGACACCAAC	(GA)_11_	214	190–416	55	GQ354549
		R: CATGCGTTTTTCACTCATGTAG	Perfect				
SPSD041	(GA)_*n*_	F: CCCAACGATGATTTCTCTTG	(GA)_26_	135	104–180	53	GQ354550
		R: TCACGGTAACACGATTAGTCC	Perfect				
SPSD042	(GA)_*n*_	F: ACCTCCCACTCGTTGCTAC	(CT)_19_	211	131–257	53	GQ354551
		R: GCCATTGCTCTGTTGTTTG	Perfect				
SPSD043	(GA)_*n*_	F: GTTCAAATGCGAACAAATCAG	(GA)_30_	277	231–252	53	GQ354552
		R: ATTCGATCTCACATGCAACAC	Perfect				
SPSD044	(GA)_*n*_	F: AGCTATATGACCCGAGACTGTG	(GA)_20_	275	254–276	55	GQ354553
		R: GGAATGGTCCCATACTTAATCC	Perfect				
SPSD045	(GA)_*n*_	F: AACGGAGGAAGTAATAAATAGC	(CT)_16_	241	214–426	53	GQ354554
		R: AGCACACACTAGCAAGGAC	Perfect				
SPSD046	(GA)_*n*_	F: CAGGTTTATCAGTGAAGACATC	(TA)_9_(GA)_12_	278	57–426	53	GQ354555
		R: GAGGTTCTTAAAGGAACATAGC	Compound				
SPSD047	(GA)_*n*_	F: CCACCTTCCTTGGATACAC	(CT)_17_	194	156–326	53	GQ354556
		R: CCACAACTACCACCTC	Perfect				
SPSD005	(AAG)_*n*_	F: TGAACCAACATAACCTACCTG	(TTC)_19_	297	192–452	55	GQ354557
		R: CCACACTAAACCGAAACTTG	Perfect				
SPSD048	(AAG)_*n*_	F: AAGGGCATAGTTTCAACCAAG	(AAG)_10_	197	88	55	GQ354565
		R: CTTTTGCTTGTTCATCAACATG	Perfect				
SPSD007	(CAG)_*n*_	F: AATCCTTCGCCTATCCTACAC	(CTG)_8_(CT)_9_	168	146–519	55	GQ354558
		R: TTCACACAGCAGACAGAACTG	Compound				
SPSD008	(CAG)_*n*_	F: GCAAGAACAGACTCAAGAGC	(CAG)_9_(CAA)_4_(CAG)_4_(CAA)_2_	276	144–309	55	GQ354559
		R: CTGCTGCTGAAGTAAAAGTTG	Compound				
SPSD049	(CAG)_*n*_	F: TGGATTGTTTCCTGATACTCCA	(TTG)_5_(CTG)_5_	299	303–667	53	GQ354560
		R: CCATAAATTGCTGCATTATTCC	Compound				
SPSD050	(CAG)_*n*_	F: GAAGCAGAAAACACAGTATTGC	(CTG)_5_	225	225–505	53	GQ354561
		R: TTGCTGGAATTTAACCTATCTG	Perfect				
SPSD053	(CAG)_*n*_	F: ACGCCTTCTTCACTCCAAC	(CTG)_8_	208	166–316	53	GQ354562
		R: GCCACCAGTTTTCATCACC	Perfect				
SPSD056	(CAG)_*n*_	F: GTTCTCCAAAGTCTCCTCCT	(TCC)_6_TTC(TCC)_2_	197	79–230	53	GQ354563
		R: ATCTTTACCTTCCTTCTGGG	Interrupted				
